# Supplementary material for: Effects of Defective Unloading and Recycling of PCNA Revealed by the Analysis of ELG1 Mutants
Source: Int J Mol Sci. 2023 Jan 13;24(2):1568. doi: 10.3390/ijms24021568 (PMC9863317; doi:10.3390/ijms24021568)
Supplement: Supplementary file 1 [file ijms-24-01568-s001.zip › ijms-2111516-supplementary.pdf]

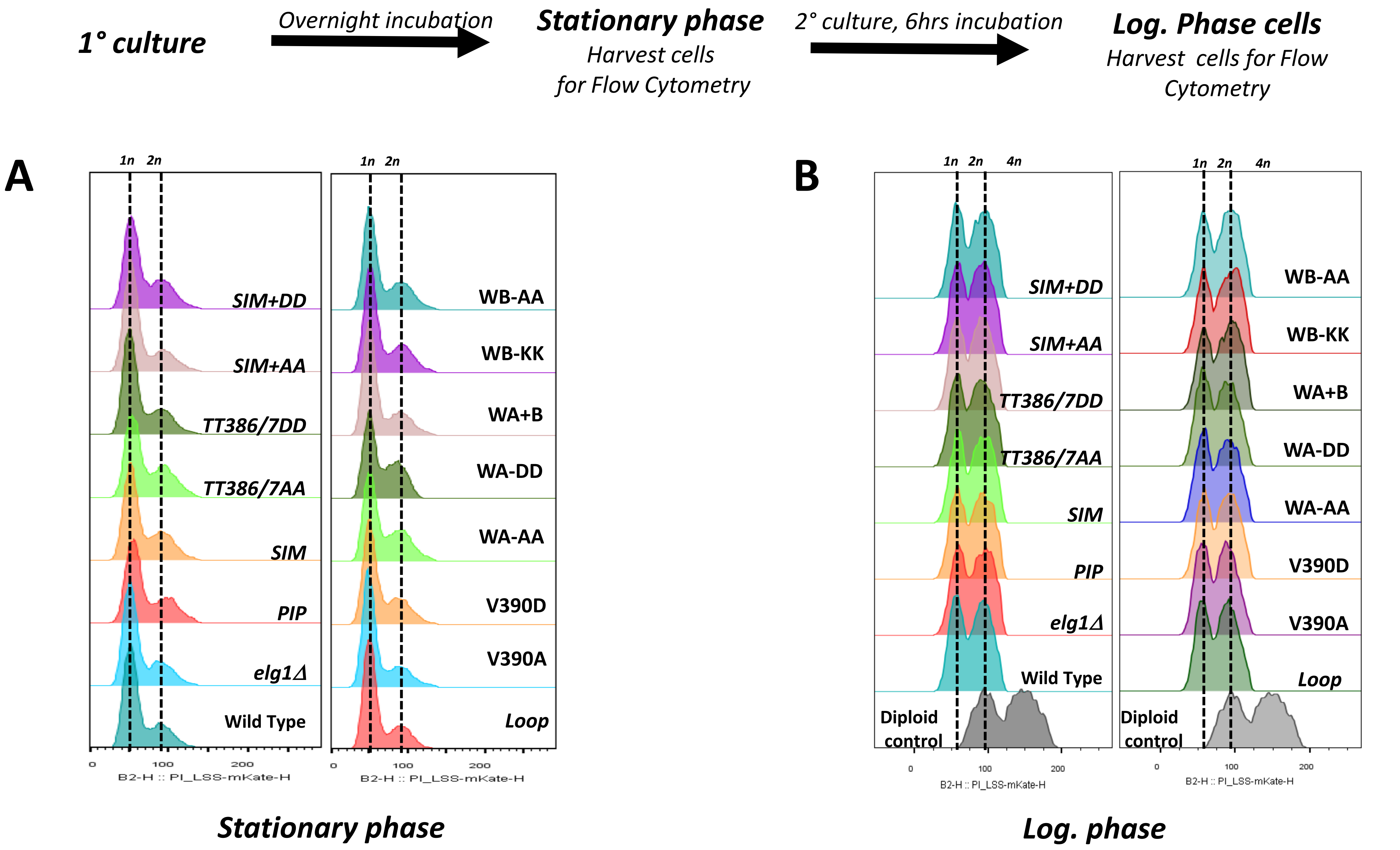

1° culture
Overnight incubation
Stationary phase
Harvest cells
for Flow Cytometry
2° culture, 6hrs incubation
Log. Phase cells
Harvest cells for Flow
Cytometry
A
1n
2n
SIM+DD
SIM+AA
TT386/7DD
TT386/7AA
SIM
PIP
elg1Δ
Wild Type
WB-AA
WB-KK
WA+B
WA-DD
WA-AA
V390D
V390A
Loop
0
100
200
B2-H :: PI_LSS-mKate-H
Stationary phase
B
4n
Diploid
control
Log. phase

Figure S1

Figure S1: Cell cycle distribution of yeast cells carrying the various *ELG1* alleles on centromeric plasmids. **A.** Stationary phase cells. **B.** Mid-logarithmic phase.

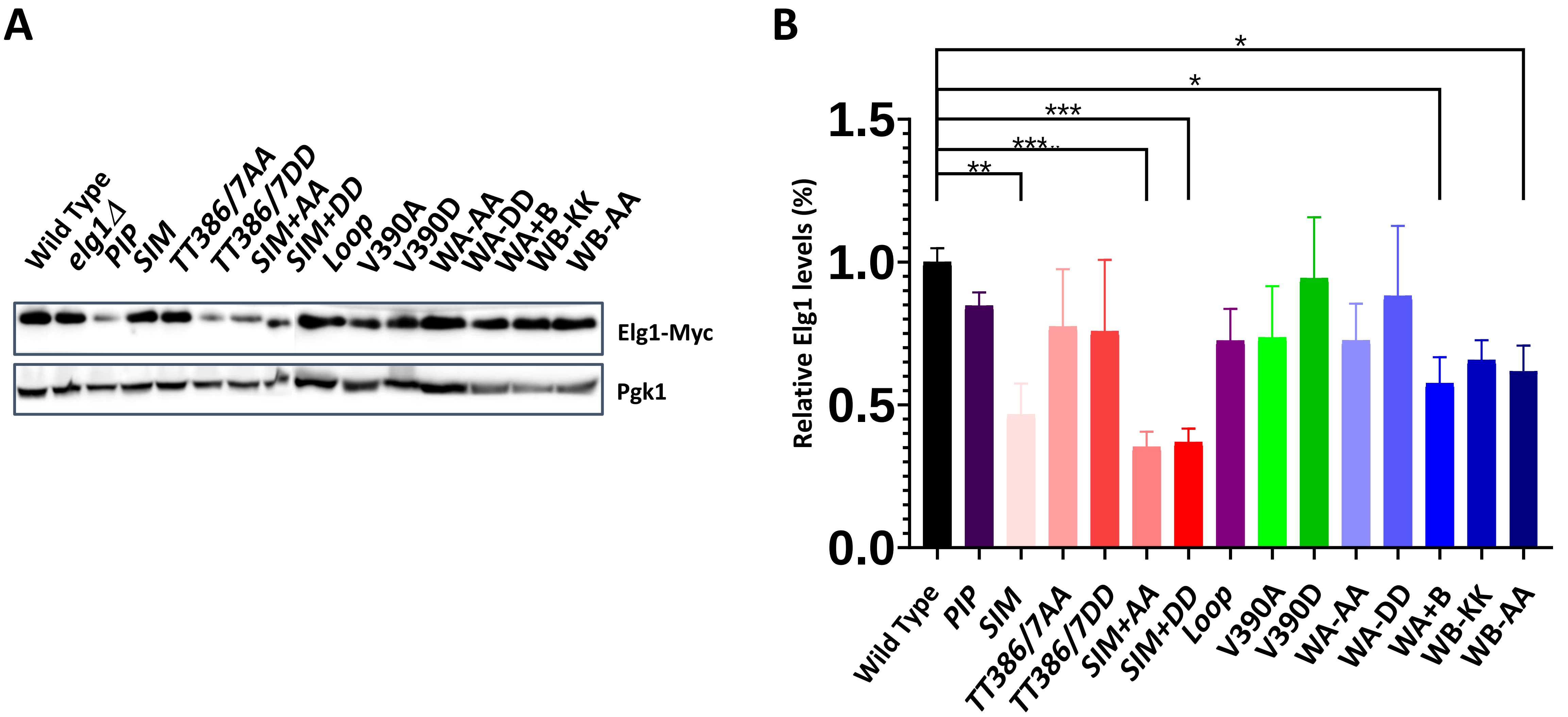
A
Wild Type
elg1Δ
PIP
SIM
TT386/7AA
TT386/7DD
SIM+AA
SIM+DD
Loop
V390A
V390D
WA-AA
WA-DD
WA+B
WB-KK
WB-AA
Elg1-Myc
Pgk1
B
Relative Elg1 levels (%)
0.0
0.5
1.0
1.5
*
*
***
***
**
Wild Type
PIP
SIM
TT386/7AA
TT386/7DD
SIM+AA
SIM+DD
Loop
V390A
V390D
WA-AA
WA-DD
WA+B
WB-KK
WB-AA

Figure S2

- Figure S2: Expression level of Elg1 mutants. **A.** Representative Western blot of mid-log cells carrying the different *ELG1* mutants. **B.** Quantitation of at least 3 such blots. * = $p\leq0.05$; ** = $p\leq0.01$; *** = $p\leq0.001$

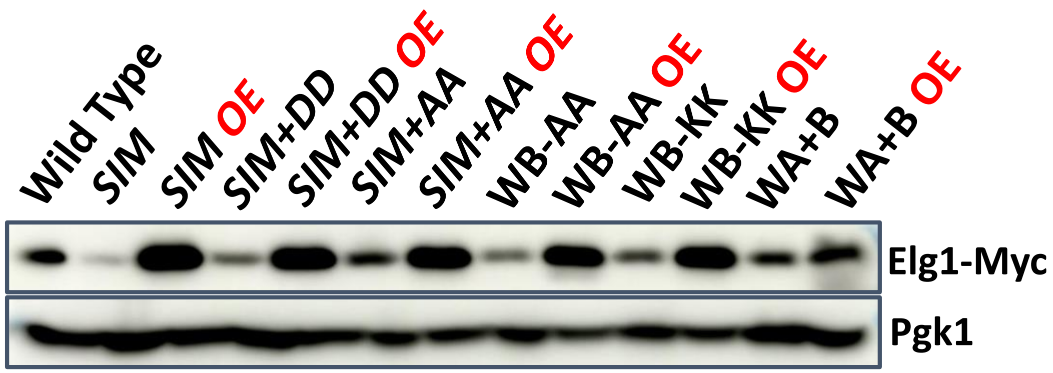

Wild Type
SIM
SIM OE
SIM+DD
SIM+DD OE
SIM+AA
SIM+AA OE
WB-AA
WB-AA OE
WB-KK
WB-KK OE
WA+B
WA+B OE
Elg1-Myc
Pgk1

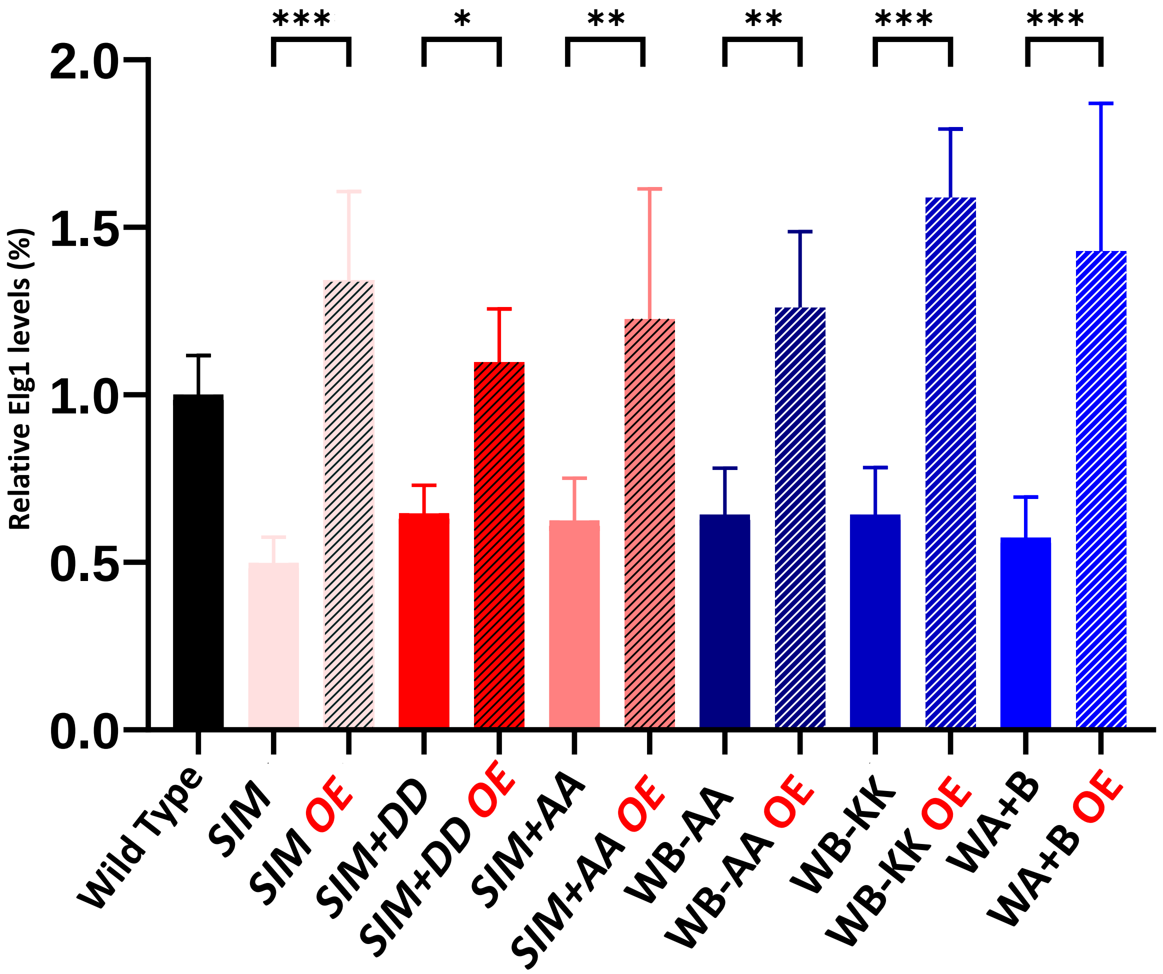

***
*
**
**
***
***
2.0
1.5
1.0
0.5
0.0
Relative Elg1 levels (%)
Wild Type
SIM
SIM OE
SIM+DD
SIM+DD OE
SIM+AA
SIM+AA OE
WB-AA
WB-AA OE
WB-KK
WB-KK OE
WA+B
WA+B OE

Figure S3

- Figure S3. Overexpression of *ELG1* alleles with low expression level restores normal protein levels. ns: not significantly different. * = $p \leq 0.05$; ** = $p \leq 0.01$; *** = $p \leq 0.001$

A

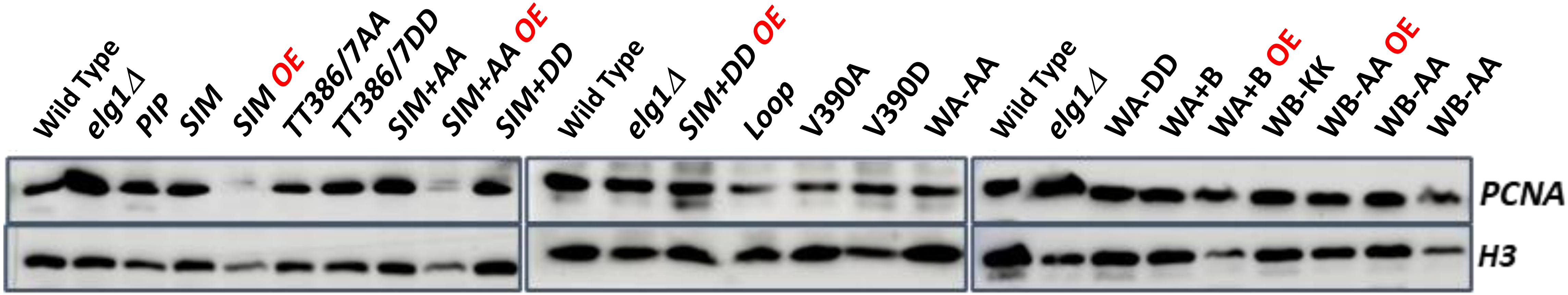
Wild Type
elg1Δ
PIP
SIM
SIM OE
TT386/7AA
TT386/7DD
SIM+AA
SIM+AA OE
SIM+DD
Wild Type
elg1Δ
SIM+DD OE
Loop
V390A
V390D
WA-AA
Wild Type
elg1Δ
WA-DD
WA+B
WA+B OE
WB-KK
WB-AA OE
WB-AA
WB-AA
PCNA
H3

B

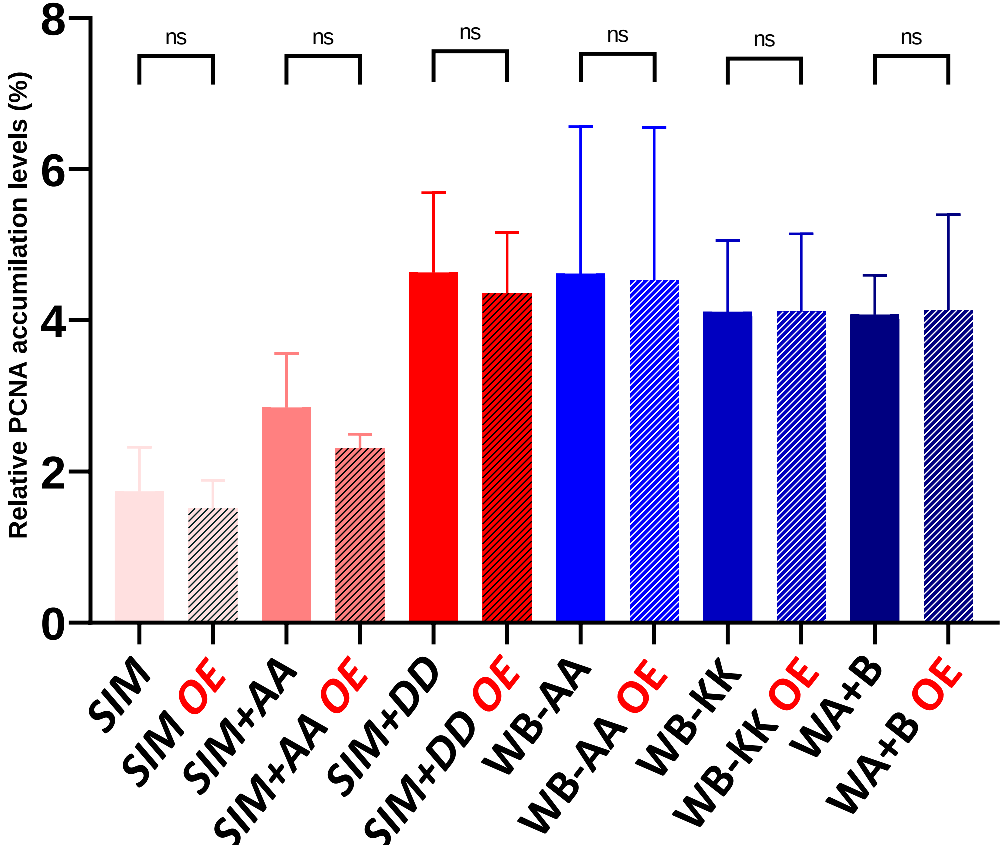
Relative PCNA accumulation levels (%)
8
6
4
2
0
ns
ns
ns
ns
ns
ns
SIM
SIM OE
SIM+AA
SIM+AA OE
SIM+DD
SIM+DD OE
WB-AA
WB-AA OE
WB-KK
WB-KK OE
WA+B
WA+B OE

Figure S4

Figure S4: **PCNA accumulation of Elg1 mutants. A.** As we checked too many mutants to be run in one western blot, we run the experiment on 3 different blot, each one containing samples from an *elg1Δ* and a WT strain. This allowed us to normalize and compare. Each of the mutants that exhibited low expression level (Figure S1) is shown with or without overexpression from a high copy number plasmid (PRS425).**B.** Quantitation of 3 western blots similar to the one seen in **A**. The expression level of the Elg1 mutants did not affect their retention on the chromatin. ns: Not significantly different.
